# Anxiety and Depression Profile Is Associated With Eating Disorders in Patients With Irritable Bowel Syndrome

**DOI:** 10.3389/fpsyt.2019.00928

**Published:** 2020-01-08

**Authors:** Chloé Melchior, Charlotte Desprez, Ghassan Riachi, Anne-Marie Leroi, Pierre Déchelotte, Najate Achamrah, Philippe Ducrotté, Marie-Pierre Tavolacci, Guillaume Gourcerol

**Affiliations:** ^1^ INSERM U1073, UNIROUEN, Normandie University, Rouen, France; ^2^ Department of Gastroenterology, Rouen University Hospital, Rouen, France; ^3^ Department of Physiology, Rouen University Hospital, Rouen, France; ^4^ Department of Nutrition, Rouen University Hospital, Rouen, France; ^5^ INSERM CIC-CRB 1404, Rouen University Hospital, Rouen, France

**Keywords:** healthy volunteers, quality of life, Rome III criteria, body mass index (BMI), stressful life events, validated self-questionnaires

## Abstract

**Objective:** To compare the prevalence of anxiety and depression states and eating disorders (EDs) between patients with irritable bowel syndrome (IBS) and healthy volunteers without IBS.

**Methods:** IBS patients according to Rome III criteria referred to our tertiary care center for therapeutic management and matched volunteers without IBS were prospectively included. EDs were screened by Sick, Control, One stone, Fat, Food—French version (SCOFF-F) questionnaire. IBS symptom severity (IBS symptom severity score), stool consistency (Bristol stool scale), anxiety and depression levels (Hospital Anxiety and Depression scale), and quality of life (validated Gastrointestinal Quality of Life Index) were assessed by validated self-questionnaires.

**Results:** IBS (228) patients and healthy volunteers (228) were included. Mean age was 42.5 ± 13.9 years with mainly women (76.7%). Among IBS patients, 25.4% had positive SCOFF-F compared to 21.1% of volunteers. IBS patients more frequently had a lower body mass index (BMI) than volunteers (p < 0.0001). IBS patients with ED had poorer quality of life and more stressful life events (p = 0.02) than IBS patients without ED. The prevalence of anxiety and depression was significantly higher in IBS patients with ED than in volunteers without ED, respectively (19.0% vs 1.9%, p=0.00, and 60.3% vs 19.7%, p < 0.0001).

**Conclusions:** The prevalence of ED assessed with positive SCOFF-F questionnaire was not significantly different between IBS patients and healthy volunteers. The combination of IBS and ED was associated with higher levels of anxiety or depression and poorer quality of life.

## Introduction

Irritable bowel syndrome (IBS) is the main functional intestinal disorder with a prevalence of about 5% in Western Europe (1). IBS is characterized by chronic abdominal pain associated with transit disorders (diarrhea, constipation, or both). The disease significantly impairs patients’ quality of life and is a significant burden on health care resources (1).

Many IBS patients consider that food is either the cause or an important trigger of their intestinal symptoms (2) and that their intestinal discomfort is related to food intolerance. This possible relationship often promotes significant changes in diet with food restriction. In some patients, restriction leads to an increased risk of undernutrition (3). The risk of food restriction is probably higher in the subgroup of IBS patients in whom IBS symptoms are associated with upper gastrointestinal (GI) symptoms, mainly functional dyspepsia but also gastro-esophageal reflux disease (3).

In clinical practice, it is sometimes difficult to determine whether this food restriction is only related to the triggering role of food intake on the onset of IBS symptoms or if it is also related to an underlying eating disorder (ED) associated with IBS. Indeed, GI symptoms are frequently reported by patients with ED (4, 5). If we consider specifically IBS, epidemiological studies have reported that 41% to 52% of ED patients also suffer from IBS (6, 7), while the severity of IBS is associated with poorer quality of life in ED patients (8). In these series, patients seemed to develop ED prior to IBS suggesting that ED may increase the risk of developing IBS (9). Whereas data exist on the prevalence of IBS symptoms in patients treated for ED, conversely the frequency of an underlying ED in patients with IBS remains poorly documented (10).

IBS is commonly associated with high anxiety and depression levels (11). ED is also associated with anxiety, depression, and other mood disorders (12). The combined presence of IBS and ED could be associated with worse treatment outcomes (13). Psychological treatments are associated with improvement in IBS symptoms as well as in quality of life in IBS (14, 15).

The aims of this prospective study were: 1) to compare the prevalence of ED between patients with IBS and healthy volunteers without IBS, matched for age and sex, and 2) to compare anxiety and depression levels according to the presence of IBS, ED, or a combination of both.

## Methods

### Design

A case–control study was carried out.

Cases had IBS (IBS+) according to Rome III criteria (16) and were 18 to 75 years old. Patients were recruited in the physiology department of our tertiary care center between 2012 and 2013. During this period, French legislation (Huriet-Sérusclat law) allowed patients to be interviewed in current care without obtaining written informed consent. The use of informatics data was declared to the Commission Nationale de I'Informatique et des Libertés (CNIL) (n° 817.917).

Controls had no IBS (IBS−), and were recruited later from the healthy volunteer registry of the Clinical Investigation Center of Rouen University Hospital in 2017. IBS− were matched on sex and age with IBS+ (1:1). Controls and IBS+ cases filled out an anonymous self-administered questionnaire comprising the SCOFF-F (Sick, Control, One stone, Fat, Food—French version) questionnaire and the Hospital Anxiety and Depression scale (HAD). IBS was assessed using Rome III criteria. Volunteers with a positive Rome III score were excluded. For healthy volunteers, in agreement with the Ethics Committee, their response to the self-questionnaire was considered as written consent. Subjects who did not complete the questionnaire were presented with information on the study. All subjects gave their tacit informed consent. The study was approved by the Ouest III Ethics Committee (2013-AOO512-53).

No patients or controls were under the age of 16.

### Data Collection

Self-reported height and weight were used to calculate body mass index (BMI) using the standard formula [BMI (kg/m²) = weight (kg)/height (m^2^) and classified as: underweight (BMI below 18.5); normal (BMI between 18.5 and 24.9); overweight (BMI between 25.0 and 29.9), and obese (BMI above 30) (according to Centers for Disease Control and Prevention)].

### Irritable Bowel Syndrome

IBS clinical phenotypes were characterized by validated self-questionnaires. IBS severity was quantified by the IBS symptom severity score (IBS-SSS) (17), and transit disorders were characterized using the Bristol stool scale from 1 to 7 (18). IBS-SSS is composed of five questions: 1) How severe is your abdominal pain? 2) Please enter the number of days that you get the pain every 10 days. 3) How severe is your abdominal distension? 4) How satisfied are you with your bowel habits? 5) Please indicate how much your IBS is affecting or interfering with your life in general. Each of the five questions generates a maximum score of 100 using prompted visual analog scales, leading to a total possible score of 500. Quality of life was assessed using the validated 36-item Gastrointestinal Quality of Life Index (GIQLI), with a maximum score of 144 (19). Items are about symptoms, physical status, emotions, social dysfunction, and effects of medical treatment. In addition, IBS patients were questioned about a possible history of acute gastroenteritis prior to onset of IBS, suggestive of post-infectious IBS. A stressful life event (as sexual abuse) prior to the beginning of symptoms was reported.

### Eating Disorders

The self-administered French version of the SCOFF questionnaire (SCOFF-F) (20) was used as a screening test for ED. This screening test does not allow a diagnosis and does not distinguish between different EDs. The score is composed of ﬁve dichotomous questions. One point is given for each “yes” answer. At least two positive answers indicate a positive SCOFF score with a sensitivity of 88.2% and a specificity of 92.5% (21). ED prevalence could be overestimated using this test.

### Anxiety and Depression

Levels of anxiety and depression were calculated using the HAD scale (22, 23) with a score of 10 out of 21 defining anxiety and depression. Patients were divided into four groups for depression and anxiety analysis: IBS+ with positive SCOFF-F (IBS+/ED+), IBS+ with negative SCOFF-F (IBS+/ED−), IBS− with positive SCOFF-F (IBS−/ED+) and IBS− with negative SCOFF-F (IBS−/ED−).

### Statistical Analysis

Data are expressed as percentage (95% confidence interval) and mean ± SD. Characteristics were compared using Fisher’s exact test for qualitative variables and Student t test for continuous variables. Fisher’s test was used to compare groups of unequal size when variances were not different. Associations were considered statistically significant when p < 0.05. The analysis was carried out using XlstatBiomed 19.5 2017.

## Results

### Characteristics of IBS+ and IBS−

A total of 456 adults between 18 and 75 years old were included, with 228 IBS+ and 228 IBS−: 53 men and 175 women in each group. Mean age was 42.5 ± 13.9 years.

In IBS+, the mean Bristol stool scale score was 4.4 ± 1.8. IBS was post-infectious in 14.9% and occurred after a stressful life event in 64.5%. The mean IBS-SSS was 248.9 ± 101.3, and quality of life was altered with a mean GIQLI score of 78.9 ± 21.8.

### Comparisons of IBS+ and IBS−

SCOFF-F questionnaire was positive in 25.4% (20.0–31.7) of IBS+ and in 21.1% (16.1–27.0) of IBS− (p = 0.27) (**Table 1**). A positive SCOFF-F was more frequent in women than in men, 29.4% and 15.1% respectively, (p = 0.02). A low BMI was more frequent in IBS+ than in IBS− while normal BMI was more frequent in volunteers than in IBS patients (p < 0.0001, **Table 1**). Prevalence of anxiety and depression was significantly higher in IBS+ [43% (36.5–49.7) and 14.0% (9.9–19.4) respectively] than in IBS− [24.7% (19.0–31.5) and 4.0% (1.9–8.1)] (<10^−3^) (**Table 1**).

**Table 1:** Characteristics of the 228 IBS+ and 228 IBS−.

	IBS + n = 228	IBS− n = 228	p
**BMI (%)**			<0.0001
**<18.5**	15.7	1.3	
**18.5–24.9**	50.9	64.0	
**25–29.9**	17.5	24.1	
**≥30**	15.8	10.5	
Positive SCOFF-F (%)	25.4	21.1	0.27
Male	17.0	13.2	0.59
Female	28.0	23.4	0.33
**Anxiety (%)**	43.0	24.7	<0.0001
**Depression (%)**	14.0	4.0	<0.0001

BMI, body mass index; IBS, irritable bowel syndrome; SCOFF-F, the French version of SCOFF (Sick, Control, One stone, Fat, Food).

### Comparison of IBS+ With or Without ED (IBS+/ED+ Vs IBS+/ED−)

Age, gender, and symptomatic IBS profile were not significantly different between IBS+ with positive or negative SCOFF-F (**Table 2**). IBS patients with positive SCOFF-F had a significantly poorer quality of life and more stressful life events (p = 0.02 and p = 0.02) (**Table 2**).

**Table 2:** Comparison between IBS+ with positive and negative SCOFF-F.

	IBS+/ED+ (n = 58)	IBS+/ED− (n = 170)	p
Age (years) mean (SD)	42.4 (14.6)	42.9 (14.5)	0.80
Female (%)	84.5	74.1	0.15
Acute gastroenteritis prior to onset of IBS (%)	12.1	15.9	0.67
**Stressful life events (%)**	**77.6**	**60.0**	**0.02**
IBS-SSS mean (SD)	264.5 (106.3)	243.4 (99.2)	0.19
**GIQLI mean (SD)**	**73.1 (19.7)**	**80.8 (22.2)**	**0.02**

IBS-SSS, IBS symptom severity score; GIQLI, validated Gastrointestinal Quality of Life Index; IBS+/ED+, IBS with positive SCOFF-F; IBS+/ED−, IBS with negative SCOFF-F.

### Comparison of Depression and Anxiety Prevalence According to the Presence of IBS, ED, or Both

**Figure 1:**
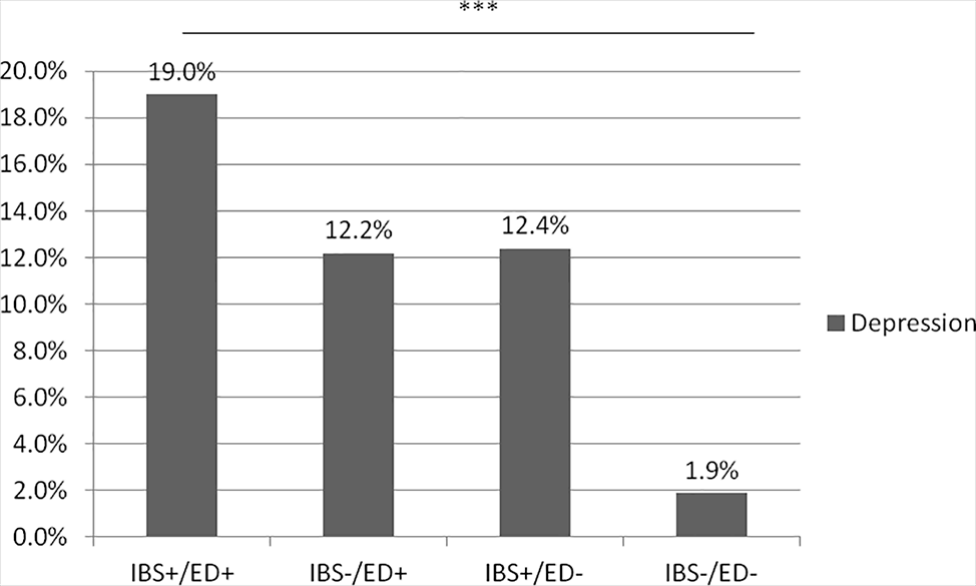
Prevalence of depression according to IBS and SCOFF-F status. IBS+/ED+, IBS with positive SCOFF-F; IBS+/ED-, IBS with negative SCOFF-F; IBS-/ED-, Volunteers without IBS with negative SCOFF-F; IBS-/ED+, Volunteers without IBS with positive SCOFF-F.

**Figure 2:**
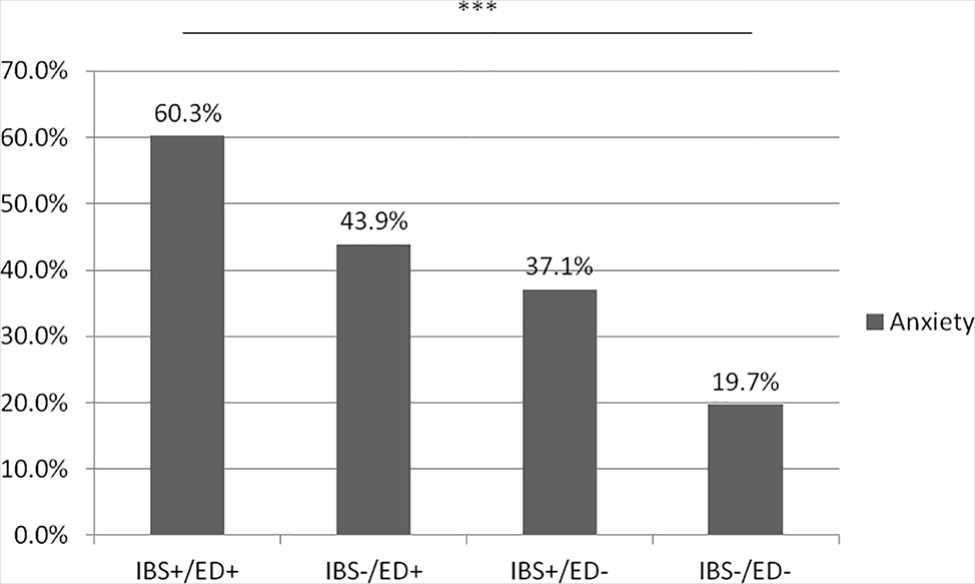
Prevalence of anxiety according to IBS and SCOFF-F status. IBS+/ED+, IBS with positive SCOFF-F; IBS+/ED-, IBS with negative SCOFF-F; IBS-/ED-, Volunteers without IBS with negative SCOFF-F; IBS-/ED+, Volunteers without IBS with positive SCOFF-F.

In our study, we analyzed four groups: IBS+/ED+ (n = 58), IBS+/ED− (n = 170), IBS−/ED+ (n = 48), and IBS−/ED− (n = 180). The prevalence of anxiety and depression was significantly higher in IBS+/ED+ than in IBS−/ED−, respectively (19.0% vs 1.9%, p = 0.0003, **Figure 1**, and 60.3% vs 19.7%, p < 0.0001, **Figure 2**). There was no difference for anxiety and depression between IBS+/ED− and IBS−/ED+.

## Discussion

To our knowledge, this is the first study, conducted in a large IBS population, highlighting a similar prevalence of SCOFF-F screened ED between IBS patients and healthy volunteers. The presence of ED in IBS is associated with the risk of a previous stressful life event. However, the SCOFF-F questionnaire, selected in this study for practical reasons because it is simple, self-administered, and easy to use, is a tool mainly for the detection of patients at risk of an ED, but it is not a test for the formal diagnosis of an ED. Nevertheless, we have previously demonstrated a correlation between positive SCOFF-F and the criteria of the *Diagnostic and Statistical Manual of Mental Disorders (DSM)-IV* which is the validated diagnostic classification for ED (20).

Our results are consistent with previous data reported in students (24) showing no difference in the prevalence of ED between IBS patients and healthy volunteers. This result suggests that the prevalence of ED is not increased in IBS patients.

Our IBS population, with a predominance of women and middle-aged patients, is comparable to that of published series. In IBS patients, age, gender, and symptomatic IBS profile were not predictive of a possible underlying ED. The lack of correlation between age, gender, and risk of ED is an unexpected result since young women are recognized as having an increased risk of ED, at least in a population of students (25). The well-established overrepresentation of women in the IBS population and the low number of young patients in our series could explain this lack of correlation. Nevertheless, in our IBS patients, high levels of anxiety or depression and poor quality of life were associated with the presence of ED. These results are in accordance with those already reported in patients with ED (26). In this latter population, both high anxiety levels and psychological abnormalities are common and associated with functional GI disorders, particularly IBS (5, 6). In ED patients, the presence of IBS was also correlated with poorer quality of life (8).

Our study did not allow us to explain why the prevalence of ED was similar in IBS+ and IBS− populations. Indeed, studies of patients with ED have reported that an ED in childhood increases the risk of further development of IBS (27). In the Perkins’ study, most patients were treated for an ED prior to IBS, with a mean delay of 10 years between the ED and the onset of IBS (9).

Nevertheless, we have demonstrated that IBS patients with ED had impaired mental health with higher levels of depression and anxiety than healthy volunteers. This result is in accordance with observations made separately in IBS and ED studies. Psychopathological profiles have already been associated with the presence of digestive symptoms in ED (5, 6). IBS patients are well known to have higher levels of anxiety and depression than controls (11, 28). Anxiety and depression are able to increase GI symptoms (29). In particular, depression has been shown to increase postprandial symptoms (29). In these IBS and ED populations and especially in patients with a combination of both IBS and ED, anxiety and depression should be systematically suspected and treated to allow better patient outcomes.

Our study has several important weaknesses: the SCOFF-F questionnaire did not allow the complete characterization of ED; there was a lack of prospective identification of IBS patients who self-imposed severe food restriction, and a possible overestimation of any association between ED and IBS as our results were obtained in a tertiary care center. Nevertheless, its strength lies in the fact that our data are based on a large cohort of IBS patients.

The present study serves as a warning not to overlook an underlying ED, especially in IBS patients who are anxious or depressed and who report previous stressful life events. This seems particularly important in clinical practice when some regimens (i.e. low-FODMAP diet, gluten-free diet) are increasingly discussed as a first-line therapeutic option in IBS management (30). Indeed in a recent IBS study, greater adherence to a low-Fermentable Oligo-, Di-, Mono-saccharides And Polyols (FODMAP) diet was associated with a positive SCOFF (31). Further investigation is required to explore suitable therapeutic options for these patients with a combination of IBS and underlying EDs associated with anxiety or depression and poor quality of life.

## Data Availability Statement

The datasets generated for this study are available on request to the corresponding author.

## Ethics Statement

The study was approved by the Ouest III Ethics Committee (2013-AOO512-53). Written informed consent for participation was not required for this study in accordance with the national legislation and the institutional requirements.

## Author Contributions

GG and M-PT designed the research. CM, CD, A-ML, PDu, M-PT, and GG performed the research. NA, PDe, and GR contributed new reagents/analytic tools. CM, CD, M-PT, and GG analyzed the data. CM, M-PT, and GG wrote the paper.

## Conflict of Interest

The authors declare that the research was conducted in the absence of any commercial or financial relationships that could be construed as a potential conflict of interest.

## Abbreviations

BMI, body mass index; ED, eating disorder; GIQLI, Gastrointestinal Quality of Life Index; HAD, Hospital Anxiety and Depression scale; IBS, irritable bowel syndrome; IBS-SSS, IBS symptom severity score; SCOFF-F, Sick, Control, One stone, Fat, Food—French version.
